# Timing of endoscopy and short-term outcomes in cirrhotic patients with acute variceal bleeding: a single-center retrospective study

**DOI:** 10.1186/s12876-026-05133-5

**Published:** 2026-07-20

**Authors:** Weixu Chen, Zhiyuan Lu, Hai Wu, Ruihua Shi

**Affiliations:** 1 Department of Gastroenterology, The Xuzhou School of Clinical Medicine of Nanjing Medical University, Xuzhou, Jiangsu People’s Republic of China; 2 Department of Gastroenterology, Xuzhou Central Hospital, Xuzhou, People’s Republic of China; 3 School of Medicine, Southeast University, Nanjing, People’s Republic of China; 4 Nanjing Medical University, Nanjing, Jiangsu People’s Republic of China; 5 Department of Gastroenterology, Zhongda Hospital, School of Medicine, Southeast University, Nanjing, People’s Republic of China

**Keywords:** Acute variceal bleeding, Endoscopy timing, Cirrhosis, Clinical outcomes

## Abstract

**Background:**

The optimal timing of endoscopy for acute variceal bleeding (AVB) remains uncertain in real-world clinical practice. This study aimed to evaluate the association between endoscopy timing and short-term outcomes in patients with AVB.

**Methods:**

We retrospectively included cirrhotic patients with endoscopically confirmed AVB admitted between January 2020 and April 2025 who underwent endoscopic therapy during hospitalization. Patients were categorized according to time from admission to endoscopy: <24 h, 24–72 h, and ≥ 72 h. The primary outcomes were 6-week mortality and rebleeding. Secondary outcomes included length of hospital stay, hospitalization cost, and transfusion requirements. Multivariable logistic regression, Firth penalized logistic regression, subgroup analyses, and sensitivity analyses were performed.

**Results:**

A total of 213 patients were included. Compared with endoscopy within 24 h, endoscopy at 24–72 h was associated with higher odds of 6-week mortality (aOR 21.7, 95% CI 2.50–390, *p* = 0.003) and rebleeding (aOR 3.35, 95% CI 1.12–11.0, *p* = 0.036). Endoscopy at ≥ 72 h was associated with higher odds of 6-week mortality in the main model (aOR 9.77, 95% CI 1.12–172, *p* = 0.038), but this association was less consistent across sensitivity analyses. Sensitivity analyses generally supported the association observed for the 24–72 h group. For secondary outcomes, delayed endoscopy was associated with longer hospital stay, whereas endoscopy at 24–72 h was associated with lower transfusion requirements.

**Conclusions:**

Endoscopy timing was associated with short-term outcomes in patients with AVB. Endoscopy at 24–72 h was associated with higher odds of 6-week mortality and rebleeding compared with endoscopy within 24 h, whereas findings for endoscopy beyond 72 h were less consistent across sensitivity analyses. These findings support individualized endoscopy timing after adequate resuscitation and stabilization.

**Supplementary Information:**

The online version contains supplementary material available at https://doi.org/10.1186/s12876-026-05133-5.

## Introduction

Acute variceal bleeding (AVB) is one of the most severe complications of cirrhosis and represents a life-threatening gastrointestinal emergency closely associated with portal hypertension [1, 2]. Despite advances in pharmacologic therapy, endoscopic techniques, and comprehensive management strategies, AVB continues to be associated with high short-term mortality and rebleeding rates and remains a major cause of cirrhosis-related death [3]. Previous studies have reported that 6-week mortality in patients with AVB ranges from 15% to 25%. Even under standard management, short-term outcomes remain suboptimal; therefore, 6-week mortality has been widely used as a key endpoint for evaluating treatment efficacy in the acute phase [4]. Improving early management to achieve better short-term outcomes remains an important clinical challenge.

Current management of AVB emphasizes early hemodynamic resuscitation, prompt use of vasoactive agents and antibiotics, and endoscopic hemostasis as the cornerstone for bleeding control and prevention of rebleeding [3, 5, 6]. Endoscopic intervention allows direct control of active bleeding and reduces the risk of early rebleeding through techniques such as band ligation, sclerotherapy, or tissue adhesive injection, making it a central component of acute management [7]. Accordingly, multiple international guidelines recommend performing endoscopy as early as possible after adequate resuscitation and consider it a standard strategy in the management of AVB [1, 3, 8].

However, the optimal timing of endoscopy remains uncertain. Most guidelines recommend that endoscopy be performed within 12 h of bleeding onset, but this recommendation is largely based on expert consensus rather than high-quality randomized controlled trials [1, 3, 8]. Previous retrospective studies and systematic reviews have used varying definitions of “urgent” or “early” endoscopy, and their findings have been inconsistent. Some studies suggest that delayed endoscopy is associated with an increased risk of rebleeding or death [7, 9], whereas others have reported that, in patients who are initially stabilized with resuscitation and medical therapy, performing endoscopy within 12–24 h is not associated with worse short-term outcomes compared with urgent endoscopy within 12 h [10, 11]. Recent meta-analyses have also failed to demonstrate a clear survival benefit of very early endoscopy, such as endoscopy within 6 h, suggesting that “the earlier, the better” may not apply to all patients and that the optimal timing remains to be defined [11].

In real-world clinical practice, particularly in primary or resource-limited settings, implementing guideline-recommended endoscopy within 12 h is often challenging. The availability of experienced endoscopists, access to anesthesia, conditions for emergency intubation, intensive care unit (ICU) bed availability, and in-hospital workflow can all influence the timing of endoscopy, leading to a gap between guideline recommendations and actual practice [1, 8]. Most existing studies on endoscopy timing have focused on whether the procedure is performed within 12 h, whereas data on patients undergoing endoscopy at 24–72 h or beyond 72 h, including their short-term outcomes and resource utilization, remain limited [12–14].

In this study, we conducted a single-center retrospective analysis to evaluate the association between endoscopy timing and short-term outcomes in patients with AVB. Unlike most previous studies that focused on dichotomous comparisons between urgent and early endoscopy, we adopted a pragmatic three-group timing strategy: early (< 24 h), intermediate (24–72 h), and delayed (≥ 72 h) endoscopy. This approach allowed us to characterize patients undergoing endoscopy beyond the conventional early window, who are frequently encountered in real-world clinical practice but remain less well described in the literature. We compared 6-week mortality and rebleeding, as well as resource-related outcomes including length of hospital stay, hospitalization costs, and transfusion requirements.

## Methods

### Study design

We retrospectively reviewed the electronic medical records of patients admitted to our hospital between January 2020 and April 2025. A total of 318 patients hospitalized for AVB who underwent endoscopic evaluation were initially identified. Among these, patients who did not receive endoscopic therapy during the index hospitalization were excluded.

The inclusion criteria were as follows: (1) age ≥ 18 years; (2) presentation with acute upper gastrointestinal bleeding (AUGIB) and endoscopic confirmation of AVB; (3) a confirmed diagnosis of cirrhosis based on clinical features, imaging findings, or histopathological evidence; (4) complete clinical and follow-up data available.

The exclusion criteria were as follows: (1) endoscopic treatment for AVB within the previous 3 months; (2) presence of end-stage disease of other major organs (such as severe heart failure, chronic obstructive pulmonary disease, or malignancies other than hepatocellular carcinoma (HCC)); (3) no endoscopic therapy performed during the index hospitalization (e.g., band ligation, sclerotherapy, or tissue adhesive injection); (4) loss to follow-up within 6 weeks after the AVB episode.

This study was approved by the Ethics Committee of Xuzhou Central Hospital. Given the retrospective nature of the study and the use of de-identified data, the requirement for written informed consent was waived.

### Data collection

The following clinical data were collected: age, sex, etiology of cirrhosis, hemoglobin level at admission, white blood cell count, platelet count, serum albumin, total bilirubin, serum creatinine, prothrombin time (PT), and international normalized ratio (INR). In addition, total length of hospital stay, total hospitalization cost, time from admission to endoscopy, ICU admission, presence of HCC, prior splenectomy, use of antibiotics, pre-endoscopy transfusion volume, total transfusion volume during hospitalization, and endoscopic treatment modalities were recorded. Child–Pugh class and the classic Model for End-Stage Liver Disease (MELD) score were calculated based on clinical history and laboratory findings. The MELD score was calculated using serum bilirubin, serum creatinine, and INR, without incorporation of serum sodium or albumin [15–17].

### Endoscopic procedure

Patients with suspected AVB were initially evaluated by a gastroenterologist in the emergency department, who determined whether admission to a general ward or ICU was required. After admission, all patients immediately received vasoactive therapy with somatostatin or octreotide, together with proton pump inhibitors and fluid resuscitation. Antibiotics were administered at the discretion of the treating physicians; among patients who received antibiotics, treatment was initiated immediately after admission. Blood transfusion was administered when either of the following criteria was met: hemoglobin < 70 g/L, or active bleeding with hemodynamic instability.

The timing of endoscopy was determined by the treating physician based on the patient’s age, mental status, preferences, and hemodynamic condition. All endoscopic procedures were performed by experienced endoscopists. For ICU patients, endoscopy was performed at the bedside under endotracheal intubation, whereas patients in the general ward underwent endoscopy under sedation in the endoscopy unit.

For esophageal variceal bleeding, endoscopic variceal ligation (EVL) or endoscopic injection sclerotherapy (EIS) was generally performed. For gastric variceal bleeding, endoscopic variceal obturation (EVO) using tissue adhesive was used. Combined endoscopic therapy was defined as receipt of at least two of the following modalities during the index endoscopic procedure: EVL, EIS, and EVO. Endoscopic hemostasis was performed in the presence of any of the following: (a) active variceal bleeding (spurting or oozing); (b) stigmata of recent bleeding, such as a white nipple sign or adherent clot; (c) blood present in the upper gastrointestinal tract with varices identified as the only potential bleeding source; or (d) hematemesis without visible blood in the gastrointestinal lumen, with varices considered the only potential bleeding source.

### Definitions

Endoscopy timing was defined as the interval from hospital admission to the initiation of endoscopy. Patients were categorized into three groups based on this interval: early (< 24 h), intermediate (24–72 h), and delayed (≥ 72 h). Pre-endoscopy transfusion was defined as any red blood cell transfusion administered before the initiation of endoscopy, and its volume was measured as the number of red blood cell units transfused before endoscopy. Total transfusion volume was defined as the total number of red blood cell units transfused during hospitalization.

### Outcome assessment and follow-up

The primary outcomes were 6-week rebleeding and 6-week mortality. Six-week rebleeding was defined as recurrent upper gastrointestinal bleeding within 6 weeks after the index AVB episode and after initial bleeding control, indicated by recurrent hematemesis, melena, or bloody stool, and accompanied by any of the following: hospital readmission, repeat endoscopic evaluation or intervention, or additional blood transfusion for suspected or confirmed variceal rebleeding. Secondary outcomes included length of hospital stay, total hospitalization cost, and total transfusion volume during hospitalization. All patients were followed up for 6 weeks after discharge.

### Statistical analysis

All statistical analyses were performed using R software (version 4.4.0; R Foundation for Statistical Computing, Vienna, Austria). Continuous variables were expressed as mean ± standard deviation or median (interquartile range [IQR]), as appropriate, and compared using one-way analysis of variance or the Kruskal–Wallis test. Categorical variables were expressed as counts (percentages) and compared using the Pearson χ² test or Fisher’s exact test.

Multivariable logistic regression was used to analyze 6-week rebleeding. Given the small number of death events, 6-week mortality was analyzed using Firth penalized logistic regression to reduce small-sample bias. Multivariable models for the primary outcomes were adjusted for age, Child–Pugh class, presence of HCC, and ICU admission. Estimates from multivariable models were reported as adjusted odds ratios (aORs) with 95% confidence intervals (CIs).

Several subgroup and sensitivity analyses were performed. A subgroup analysis was conducted among patients who were not admitted to the ICU during the index hospitalization. Sensitivity analyses included additional adjustment for pre-endoscopy transfusion volume, replacement of Child–Pugh class with MELD score as a measure of liver disease severity, restriction of the cohort to patients who underwent endoscopy within 72 h (< 24 h vs. 24–72 h), and additional guideline-based timing analyses using four categories (≤ 12 h, 12–24 h, 24–72 h, and ≥ 72 h) and a simplified three-category classification (≤ 12 h, 12–24 h, and > 24 h). In addition, inverse probability of treatment weighting (IPTW) was performed using stabilized weights estimated from a multinomial propensity score model. Covariate balance before and after IPTW was assessed using standardized mean differences. Because residual age imbalance remained after weighting, IPTW-weighted outcome models were additionally adjusted for age.

Secondary outcomes (length of stay, hospitalization cost, and transfusion volume) were analyzed using generalized linear models with a gamma distribution and log link function due to their skewed distributions. For transfusion volume, 0.5 was added prior to modeling to account for zero values. Results were presented as exp(β) with 95% CIs. All tests were two-sided, and *P* < 0.05 was considered statistically significant.

## Results

### Patient characteristics

**Fig. 1:**
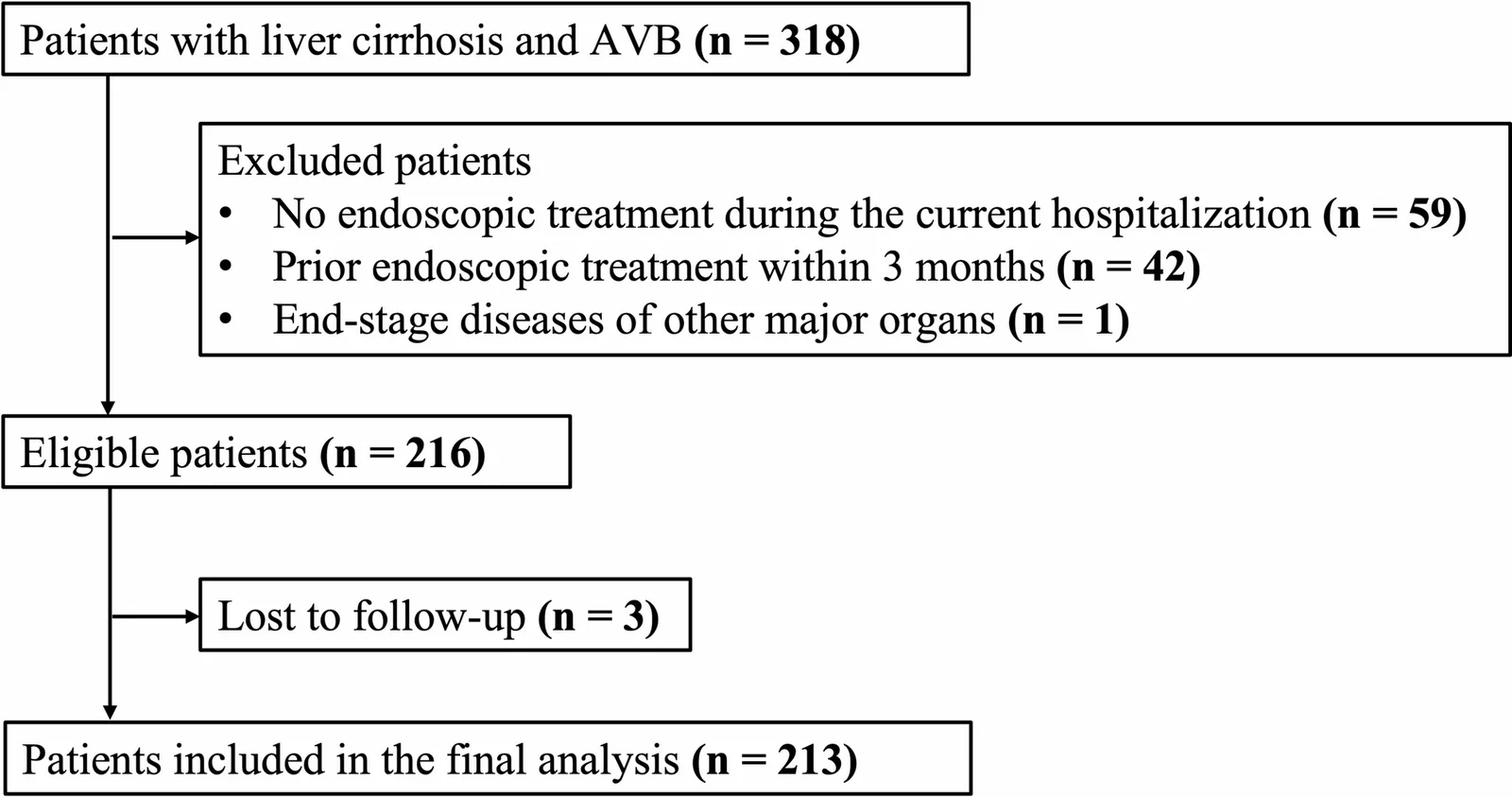
Flow chart of patient selection

Between January 2020 and April 2025, a total of 318 patients with cirrhosis and AVB were hospitalized at our center. According to the inclusion and exclusion criteria, 102 patients were excluded, including 59 patients who did not undergo endoscopic therapy during the index hospitalization, 42 patients who had received endoscopic therapy within the previous 3 months, and 1 patient with end-stage disease of other major organs. Ultimately, 216 patients met the inclusion criteria, among whom 3 were lost to follow-up within 6 weeks, leaving 213 patients included in the final analysis (Fig. 1).

Among the 213 patients included in the analysis, 45 (21.1%) underwent early endoscopy, 55 (25.8%) underwent intermediate endoscopy, and 113 (53.1%) underwent delayed endoscopy (Table 1). The mean age of the study population was 57.5 ± 11.7 years, and 152 patients (71%) were male. The most common etiology of cirrhosis was viral hepatitis (123/213, 58%). Most patients were classified as Child–Pugh class B (157/213, 74%). The median MELD score was 9.4 (IQR: 8.3–11.6). ICU admission differed significantly among the three groups and was most frequent in the early endoscopy group. Pre-endoscopy transfusion was administered to 117 patients (55%), with a median transfusion volume of 2.0 units (IQR: 0.0–4.0); both the proportion of patients receiving transfusion and the transfusion volume differed significantly across the timing groups. Endoscopic treatment modalities are summarized in Supplementary Table 1. EIS was the most frequently used endoscopic modality, followed by EVO and EVL. The distributions of EVL and EIS were similar among the three groups, whereas EVO and combined endoscopic therapy differed significantly across the timing groups.

**Table 1:** Baseline characteristics according to timing of endoscopy

Characteristic	All patients	Early endoscopy	Intermediate endoscopy	Delayed endoscopy	*P* value
	*N* = 213	(< 24 h)	(24–72 h)	(≥ 72 h)	
		*N* = 45	*N* = 55	*N* = 113	
Age (years)	57.5 ± 11.7	56.0 ± 10.3	57.2 ± 11.6	58.3 ± 12.4	0.5
Sex (male)	152 (71%)	34 (76%)	44 (80%)	74 (65%)	0.12
Etiology					**0.046**
Virus	123 (58%)	28 (62%)	39 (71%)	56 (50%)	
Alcohol	36 (17%)	10 (22%)	8 (15%)	18 (16%)	
AIH	14 (6.6%)	3 (6.7%)	2 (3.6%)	9 (8.0%)	
Other	40 (19%)	4 (8.9%)	6 (11%)	30 (27%)	
Laboratory tests					
Hemoglobin (g/L)	80.1 ± 23.2	77.8 ± 24.4	84.4 ± 23.4	78.9 ± 22.6	0.2
White blood cell (×10^9^/L)	6.5 (4.3–8.7)	5.8 (4.0-8.9)	6.8 (4.8–8.3)	6.7 (4.1–8.7)	0.8
Platelet count (×10^9^/L)	114.0 (72.0-177.0)	112.0 (76.0-170.0)	115.0 (80.0-173.0)	112.0 (65.0-188.0)	> 0.9
Prothrombin time (seconds)	14.8 ± 2.7	15.6 ± 3.4	14.7 ± 1.7	14.5 ± 2.8	**0.025**
International normalized ratio (INR)	1.2 (1.2–1.4)	1.3 (1.2–1.4)	1.2 (1.1–1.3)	1.2 (1.2–1.4)	**0.037**
Total bilirubin (µmol/L)	16.1 (12.3–23.8)	21.0 (14.9–33.1)	14.9 (11.5–21.5)	17.0 (13.1–25.0)	**0.006**
Albumin (g/L)	30.2 ± 5.5	28.7 ± 5.4	31.5 ± 4.8	30.1 ± 5.8	**0.011**
Serum creatinine (µmol/L)	58.6 (48.0–72.0)	53.1 (45.7–66.7)	59.0 (49.6–74.0)	60.0 (47.0–73.0)	0.2
Child-Pugh class					0.13
A	44 (21%)	4 (8.9%)	12 (22%)	28 (25%)	
B	157 (74%)	37 (82%)	40 (73%)	80 (71%)	
C	11 (5.2%)	4 (8.9%)	3 (5.5%)	4 (3.6%)	
MELD score	9.4 (8.3–11.6)	11.0 (8.7–12.3)	9.1 (7.9–11.3)	9.7 (8.3–11.2)	**0.019**
ICU admission	42 (20%)	25 (56%)	5 (9.1%)	12 (11%)	**< 0.001**
Pre-endoscopy transfusion, *n* (%)	117 (55%)	18 (40%)	25 (45%)	74 (66%)	**0.004**
Pre-endoscopy transfusion volume (units)	2.0 (0.0, 4.0)	0.0 (0.0, 4.0)	0.0 (0.0, 3.0)	2.0 (0.0, 4.0)	**0.004**
Hepatocellular carcinoma	44 (21%)	9 (20%)	14 (25%)	21 (19%)	0.6
History of splenectomy	61 (29%)	7 (16%)	19 (35%)	35 (31%)	0.073
Antibiotic use	100 (48%)	20 (47%)	23 (42%)	57 (51%)	0.6

Data are presented as mean ± SD, median (IQR), or *n* (%)

Bold values indicate statistical significance (*P* < 0.05)

### Clinical outcomes according to endoscopy timing

As shown in Table 2, the overall 6-week rebleeding rate was 25% (54/213), and the 6-week mortality rate was 7.0% (15/213). The 6-week rebleeding rates in the early (< 24 h), intermediate (24–72 h), and delayed (≥ 72 h) endoscopy groups were 16% (7/45), 35% (19/55), and 25% (28/113), respectively, with no significant difference among the three groups (*p* = 0.093). In contrast, the 6-week mortality rates differed significantly among the groups, at 2.2% (1/45), 15% (8/55), and 5.3% (6/113), respectively (*p* = 0.045).

**Table 2:** Clinical outcomes according to timing of endoscopy

Clinical outcomes	All patients	Early endoscopy (< 24 h)	Intermediate endoscopy (24–72 h)	Delayed endoscopy (≥ 72 h)	*P* value
	*N* = 213	*N* = 45	*N* = 55	*N* = 113	
6-week rebleeding	54 (25%)	7 (16%)	19 (35%)	28 (25%)	0.093
6-week mortality	15 (7.0%)	1 (2.2%)	8 (15%)	6 (5.3%)	**0.045**
Length of hospital stay (days)	11 (8–14)	9 (5–13)	9 (7–13)	12 (10–15)	**< 0.001**
Total hospitalization cost (CNY)	16,682 (12466–23932)	17,625 (11302–29189)	13,659 (9911–18675)	17,440 (14161–23932)	**0.007**
Total transfusion volume (units)	2.0 (0.0, 6.0)	4.0 (0.0, 10.0)	0.0 (0.0, 4.0)	3.0 (0.0, 5.5)	**0.011**

Data are presented as median (IQR) or *n* (%)

Bold values indicate statistical significance (*P* < 0.05)

For secondary outcomes, significant differences were observed among the three groups in length of hospital stay, total hospitalization cost, and total transfusion volume. The median length of hospital stay was 9 days in both the early and intermediate groups (IQR: 5–13 days and 7–13 days, respectively), which was shorter than that in the delayed group (12 days, IQR: 10–15 days) (*p* < 0.001). The median hospitalization cost in the intermediate group was 13,659 CNY (IQR: 9911–18675 CNY), which was lower than that in the early and delayed groups (17625 CNY and 17440 CNY, respectively) (*p* = 0.007). In addition, the intermediate group had the lowest median total transfusion volume (0.0 units, IQR: 0.0–4.0), compared with 4.0 units (IQR: 0.0–10.0) in the early group and 3.0 units (IQR: 0.0–5.5) in the delayed group (*p* = 0.011).

### Multivariable analyses of primary outcomes

Multivariable Firth penalized logistic regression analysis showed that endoscopy timing, liver function status, and the presence of HCC were significantly associated with 6-week mortality (Table 3). Compared with the early endoscopy group (< 24 h), both the intermediate group (24–72 h) and the delayed group (≥ 72 h) were associated with higher odds of 6-week mortality (aOR 21.7, 95% CI 2.50–390, *p* = 0.003; and aOR 9.77, 95% CI 1.12–172, *p* = 0.038, respectively). In addition, each increase in Child–Pugh class was associated with higher odds of 6-week mortality (aOR 11.9, 95% CI 2.43–68.6, *p* = 0.002). The presence of HCC was also associated with increased odds of mortality (aOR 10.0, 95% CI 3.05–39.6, *p* < 0.001). ICU admission was included in the model but was not significantly associated with 6-week mortality.

**Table 3:** Risk factors associated with 6-week mortality

	Univariable analysis, OR (95% CI)	*P* value	Multivariable analysis, aOR (95% CI)	*P* value
Timing of endoscopy				
<24 h (reference)	Reference	—	Reference	—
24–72 h	7.49 (1.30–142)	0.063	21.7 (2.50–390)	**0.003**
≥72 h	2.47 (0.41–47.3)	0.409	9.77 (1.12–172)	**0.038**
Age (years)	1.03 (0.99–1.08)	0.147	1.05 (1.00–1.11)	0.061
Child-Pugh class (per class increase)	6.27 (1.92–21.5)	**0.003**	11.9 (2.43–68.6)	**0.002**
Hepatocellular carcinoma (Yes vs. No)	9.65 (3.22–32.7)	**< 0.001**	10.0 (3.05–39.6)	**< 0.001**
ICU admission (Yes vs. No)	1.02 (0.22–3.40)	0.977	3.25 (0.62–15.6)	0.155

*OR* odds ratio, *aOR* adjusted odds ratio, *CI* confidence interval

Bold values indicate statistical significance (*P* < 0.05)

In multivariable logistic regression analysis, both endoscopy timing and the presence of HCC were significantly associated with 6-week rebleeding (Table 4). Compared with the early endoscopy group (< 24 h), the intermediate group (24–72 h) was associated with higher odds of rebleeding (aOR 3.35, 95% CI 1.12–11.0, *p* = 0.036), whereas no significant difference was observed between the delayed and early groups. The presence of HCC was also associated with higher odds of rebleeding (aOR 3.24, 95% CI 1.54–6.86, *p* = 0.002). ICU admission was not significantly associated with 6-week rebleeding.

**Table 4:** Risk factors associated with 6-week rebleeding

	Univariable analysis, OR (95% CI)	*P* value	Multivariable analysis, aOR (95% CI)	*P* value
Timing of endoscopy				
<24 h (reference)	Reference	—	Reference	—
24–72 h	2.87 (1.11–8.09)	**0.035**	3.35 (1.12–11.0)	**0.036**
≥72 h	1.79 (0.75–4.77)	0.212	2.24 (0.81–6.92)	0.136
Age (years)	1.03 (1.00–1.05)	0.059	1.03 (1.00–1.06)	0.074
Child–Pugh class (per class increase)	1.62 (0.85–3.21)	0.155	1.56 (0.76–3.26)	0.230
Hepatocellular carcinoma (Yes vs. No)	3.31 (1.63–6.70)	**< 0.001**	3.24 (1.54–6.86)	**0.002**
ICU admission (Yes vs. No)	0.76 (0.32–1.66)	0.515	1.19 (0.44–3.11)	0.719

*OR* odds ratio, *aOR* adjusted odds ratio, *CI* confidence interval

Bold values indicate statistical significance (*P* < 0.05)

### Subgroup and sensitivity analyses

A subgroup analysis was performed among patients who were not admitted to the ICU during the index hospitalization. In this non-ICU subgroup, the 6-week mortality rates were 0% in the early group, 14% in the intermediate group, and 5.0% in the delayed group, respectively, and the difference did not reach statistical significance (*p* = 0.071). The corresponding 6-week rebleeding rates were 15%, 34%, and 24.8%, respectively, with no significant difference among the three groups (*p* = 0.254) (Supplementary Table 2).

Several sensitivity analyses were performed to assess whether the findings were consistent across different model specifications (Table 5). After additional adjustment for pre-endoscopy transfusion volume, both the intermediate and delayed groups remained significantly associated with higher odds of 6-week mortality compared with the early group (aOR 23.2, 95% CI 2.59–438, *p* = 0.003; and aOR 11.0, 95% CI 1.17–210, *p* = 0.035, respectively). For 6-week rebleeding, the intermediate group remained significantly associated with higher odds of rebleeding (aOR 3.29, 95% CI 1.07–11.1, *p* = 0.044), whereas the delayed group did not. Pre-endoscopy transfusion volume itself was not significantly associated with either 6-week mortality or 6-week rebleeding (Supplementary Table 3).

**Table 5:** Sensitivity analyses of the association between endoscopy timing and primary outcomes

Clinical outcomes	Analysis	24–72 h vs. < 24 h OR/aOR (95% CI)	*P* value	≥ 72 h vs. < 24 h OR/aOR (95% CI)	*P* value
6-week mortality	Adjusted for pre-endoscopy transfusion volume	23.2 (2.59–438)	**0.003**	11.0 (1.17–210)	**0.035**
6-week mortality	MELD instead of Child–Pugh class	12.0 (1.71–161)	**0.010**	4.16 (0.60–53.2)	0.158
6-week mortality	IPTW-weighted	16.32 (1.88–141.48)	**0.012**	6.55 (0.70–61.35)	0.101
6-week mortality	Restricted to < 72 h	30.78 (2.37–1139.49)	**0.006**	-	-
6-week rebleeding	Adjusted for pre-endoscopy transfusion volume	3.29 (1.07–11.1)	**0.044**	2.27 (0.76–7.49)	0.156
6-week rebleeding	MELD instead of Child–Pugh class	3.52 (1.17–11.7)	**0.031**	2.14 (0.77–6.61)	0.162
6-week rebleeding	IPTW-weighted	3.27 (1.02–10.48)	**0.048**	2.17 (0.73–6.49)	0.166
6-week rebleeding	Restricted to < 72 h	4.00 (1.22–15.10)	**0.029**	-	-

Bold values indicate statistical significance (*P* < 0.05)

When MELD score was used instead of Child–Pugh class to adjust for liver disease severity, the intermediate group remained significantly associated with both 6-week mortality (aOR 12.0, 95% CI 1.71–161, *p* = 0.010) and 6-week rebleeding (aOR 3.52, 95% CI 1.17–11.7, *p* = 0.031). The association between delayed endoscopy and 6-week mortality was attenuated and was no longer statistically significant in this model (Supplementary Table 4).

In the IPTW-weighted analysis with additional adjustment for age, the intermediate group remained significantly associated with higher odds of 6-week mortality (OR 16.32, 95% CI 1.88–141.48, *p* = 0.012) and 6-week rebleeding (OR 3.27, 95% CI 1.02–10.48, *p* = 0.048) compared with the early group. The estimates for the delayed group were not statistically significant for either outcome. Balance diagnostics after IPTW are shown in Supplementary Tables 5, and the IPTW-weighted outcome models are shown in Supplementary Table 6.

In the sensitivity analysis restricted to patients who underwent endoscopy within 72 h, the intermediate group remained significantly associated with higher odds of both 6-week mortality (OR 30.78, 95% CI 2.37–1139.49, *p* = 0.006) and 6-week rebleeding (OR 4.00, 95% CI 1.22–15.10, *p* = 0.029) compared with the early group.

To further assess the robustness of our findings using guideline-based timing cutoffs, we performed an additional analysis using four categories: ≤12 h, 12–24 h, 24–72 h, and ≥ 72 h. The 6-week rebleeding rates were 18%, 14%, 35%, and 25%, respectively, and the corresponding 6-week mortality rates were 0%, 3.6%, 15%, and 5.3%, respectively (Supplementary Table 7). In adjusted models, the 24–72 h group showed numerically higher odds of 6-week rebleeding, although this association was not statistically significant. For 6-week mortality, the 24–72 h and ≥ 72 h groups were associated with higher odds of mortality; however, the estimates had wide confidence intervals, partly because the ≤ 12 h reference group included only 17 patients and no mortality events (Supplementary Table 8). A simplified three-category analysis using ≤ 12 h, 12–24 h, and > 24 h showed a similar pattern, with > 24 h associated with higher odds of 6-week mortality but not significantly associated with 6-week rebleeding (Supplementary Tables 9 and 10).

### Factors associated with secondary outcomes

In multivariable generalized linear model analysis, endoscopy timing showed differential associations with length of hospital stay, hospitalization cost, and transfusion volume (Table 6). Compared with the early group (< 24 h), patients in the delayed group (≥ 72 h) had a significantly longer hospital stay (ratio 1.39, 95% CI 1.15–1.71, *p* = 0.001), whereas no significant difference was observed between the intermediate and early groups. No significant association was observed between endoscopy timing and hospitalization cost.

**Table 6:** Factors associated with secondary clinical outcomes

	Hospital stay	*P* value	Hospitalization cost	*P* value	Total transfusion volume	*P* value
	Ratio (95% CI)		Ratio (95% CI)		Ratio (95% CI)	
Timing of endoscopy						
<24 h (reference)	Reference	—	Reference	—	Reference	—
24–72 h	1.18 (0.95–1.50)	0.149	0.91 (0.70–1.17)	0.455	0.64 (0.43–0.94)	**0.024**
≥72 h	1.39 (1.15–1.71)	**0.001**	0.99 (0.82–1.23)	0.942	0.81 (0.57–1.14)	0.239
Age (years)	1.00 (0.99–1.00)	0.793	1.00 (0.99–1.00)	0.226	0.99 (0.98–1.00)	**0.012**
Child–Pugh class (per class increase)	1.07 (0.89–1.29)	0.438	0.82 (0.65–1.04)	0.09	1.44 (1.04–1.99)	**0.033**
Hepatocellular carcinoma (Yes vs. No)	1.07 (0.90–1.24)	0.429	0.72 (0.55–0.90)	**0.008**	0.94 (0.67–1.34)	0.708
Albumin (g/L)	0.97 (0.96–0.99)	**< 0.001**	0.94 (0.92–0.96)	**< 0.001**	0.93 (0.91–0.96)	**< 0.001**
WBC (×10⁹/L)	1.01 (1.00–1.03)	0.095	1.02 (1.00–1.04)	0.067	1.03 (0.99–1.07)	0.096

Ratios represent exp(β)

*CI* confidence interval

Bold values indicate statistical significance (*P *< 0.05)

Regarding transfusion volume, patients in the intermediate group had significantly lower transfusion requirements compared with those in the early group (ratio 0.64, 95% CI 0.43–0.94, *p* = 0.024), whereas no significant difference was observed for the delayed group. In addition to timing, a higher Child–Pugh class was also associated with increased transfusion volume (ratio 1.44, 95% CI 1.04–1.99, *p* = 0.033).

Furthermore, lower serum albumin levels were significantly associated with longer hospital stay, higher hospitalization cost, and increased transfusion volume (all *p* < 0.001).

## Discussion

In this single-center retrospective study of patients with cirrhosis and AVB, endoscopy timing was associated with short-term clinical outcomes. Compared with endoscopy performed within 24 h after admission, endoscopy performed between 24 and 72 h was associated with higher odds of both 6-week mortality and 6-week rebleeding. These findings suggest that endoscopy timing may be an important marker of clinical course and short-term prognosis in patients with AVB in real-world practice.

International guidelines, including the Baveno VII consensus, AASLD, and ESGE, recommend early endoscopy in patients with suspected AVB after appropriate resuscitation and stabilization [1, 3, 8]. In the present study, “early endoscopy” was defined as endoscopy performed within 24 h after admission. Although this differs from the 12 h window commonly recommended in guidelines, the 24 h threshold reflects clinical practice in our center and in many real-world settings. In routine practice, the availability of experienced endoscopists, anesthesia support, and emergency airway management may be limited, particularly during nights and holidays. Patients with hemodynamic instability may require ICU admission, airway protection, or further resuscitation before undergoing endoscopy, which may delay the procedure. Although the AASLD guidance recommends upper endoscopy within 12 h of presentation in patients with acute variceal hemorrhage, evidence supporting the superiority of ≤ 12 h over 12–24 h remains limited. Several retrospective studies and meta-analyses have reported no significant difference in short-term outcomes between urgent (≤ 12 h) and early (12–24 h) endoscopy in hemodynamically stabilized patients [3, 10, 11, 18]. Therefore, the 24 h threshold used in this study provides a clinically relevant framework for evaluating endoscopy timing in real-world practice, especially in settings where immediate endoscopy is not always feasible.

We further separated patients undergoing endoscopy after 24 h into 24–72 h and ≥ 72 h groups because these windows may represent clinically distinct delayed-treatment patterns. The 24–72 h group reflects patients in whom endoscopy was delayed beyond the first day but still performed during the early hospitalization phase, whereas the ≥ 72 h group may include a more heterogeneous and selected population, including patients with temporary stabilization, delayed procedural scheduling, comorbidities, or other clinical constraints. Therefore, a guideline-based classification such as ≤ 12 h, 12–24 h, and > 24 h would not distinguish these two groups. We used categorical timing windows rather than treating timing as a continuous variable because our objective was to compare clinically meaningful real-world timing patterns rather than assume a uniform relationship between increasing delay and outcomes.

Nevertheless, to address the guideline-recommended timing thresholds, we additionally performed guideline-based analyses using four categories (≤ 12 h, 12–24 h, 24–72 h, and ≥ 72 h) and a simplified three-category classification (≤ 12 h, 12–24 h, and > 24 h). These analyses partly supported the direction of the primary findings, particularly for mortality after 24 h, but the estimates were imprecise because of the small size of the ≤ 12 h group and the absence of mortality events in this reference group. Therefore, these results should be viewed as exploratory robustness assessments rather than definitive evidence supporting a specific timing cutoff.

In the present study, patients undergoing endoscopy at 24–72 h had higher odds of both 6-week mortality and rebleeding. This timing window may represent a clinically complex subgroup of patients in whom endoscopy could not be completed within the initial 24 h, but for whom further delay was not considered clinically appropriate. Several factors may partly explain this association. First, ongoing or intermittent bleeding before definitive endoscopic therapy may increase the risk of rebleeding during the waiting period. Second, patients undergoing endoscopy in this window may have more complex clinical conditions, requiring initial stabilization, transfusion, airway evaluation, ICU assessment, or coordination of procedural resources. Therefore, the higher risk observed in the 24–72 h group may reflect both delayed hemostatic intervention and unmeasured clinical complexity. This interpretation is consistent with previous studies reporting associations between delayed endoscopy and worse outcomes. A prospective study by Chen et al. demonstrated that, in patients presenting with hematemesis and endoscopically confirmed active bleeding, endoscopy within 12 h significantly reduced 6-week rebleeding and mortality compared with procedures performed after 12 h [7]. Similarly, Hsu et al., in a retrospective study of 373 patients, identified delayed endoscopy (> 15 h) as an independent risk factor for in-hospital mortality [9]. A systematic review by Bai et al. further supported the potential benefit of early endoscopic intervention in improving short-term outcomes [19].

In contrast, the interpretation of the delayed group (≥ 72 h) is more complex. In the main multivariable model, endoscopy performed after 72 h was associated with higher odds of 6-week mortality compared with endoscopy within 24 h, and a similar association was observed after additional adjustment for pre-endoscopy transfusion volume. However, this association was less consistent across sensitivity analyses, particularly when MELD score was used instead of Child–Pugh class and in the IPTW-weighted analysis. Clinically, patients who underwent endoscopy after 72 h may represent a heterogeneous and highly selected group. Some patients may have experienced delayed definitive treatment because of severe comorbidities, resource limitations, or clinical instability, whereas others may have achieved temporary hemostasis and clinical stabilization with pharmacologic therapy and supportive care, allowing endoscopy to be deferred. Therefore, the ≥ 72 h group should not be interpreted simply as a homogeneous delayed-treatment group. Similar observations have been reported in previous studies. Peng et al. found no significant differences in 5-day hemostasis failure or in-hospital mortality across different timing windows (< 12 h, < 24 h, and < 48 h) [13]. Yoo et al., in a multicenter study, also reported no significant differences in 6-week mortality, in-hospital mortality, or rebleeding between ≤ 12 h and > 12 h groups [10]. Likewise, Nguyen et al. showed no significant differences in early versus delayed endoscopy for outcomes including treatment failure, mortality, transfusion requirements, and length of stay, even after propensity score matching [12]. Taken together, these findings suggest that the prognostic implications of delayed endoscopy may depend on patient selection, clinical stability, and the adequacy of initial medical therapy; therefore, the absence of consistently worse outcomes in the ≥ 72 h group should not be interpreted as evidence that delaying endoscopy beyond 72 h is safe in general.

The subgroup and sensitivity analyses provided further context for interpreting these findings. These analyses were particularly important because ICU admission and pre-endoscopy transfusion differed across timing groups and may reflect differences in acute clinical severity and resuscitation needs. In the non-ICU subgroup, the differences in 6-week mortality and rebleeding among the timing groups did not reach statistical significance, although the intermediate group had numerically higher event rates. After additional adjustment for pre-endoscopy transfusion volume, the association between the intermediate group and the primary outcomes was largely consistent with the main analysis. Similar findings were observed when MELD score was used instead of Child–Pugh class to adjust for liver disease severity. In the IPTW-weighted analysis, the intermediate group also remained associated with higher odds of both 6-week mortality and rebleeding, whereas the estimates for the ≥ 72 h group were not statistically significant. Taken together, these analyses support the relative consistency of the association observed for the 24–72 h group, while the association for the ≥ 72 h group appeared less stable. Nevertheless, the wide confidence intervals, particularly for mortality, indicate limited precision and support a cautious interpretation of these exploratory findings.

In addition, the relationship between endoscopy timing and outcomes should be interpreted in the context of early resuscitation and hemodynamic stabilization. In patients with AVB, circulatory instability, tissue hypoperfusion, vasopressor requirement, duration of hypotension, lactate dynamics, and the adequacy of initial resuscitation may influence both the timing of endoscopy and short-term prognosis. Although these variables were not available in our dataset, previous studies in emergency and critically ill patients have shown that early lactate dynamics and the duration below target mean arterial pressure are associated with short-term mortality [20, 21]. Therefore, the lack of detailed hemodynamic and perfusion-related variables represents an important source of potential residual confounding in the present study.

Our findings also highlight the impact of underlying disease severity on patient outcomes. The Child–Pugh score reflects the degree of hepatic decompensation, with higher classes indicating poorer hepatic reserve, while the presence of HCC often indicates advanced liver disease and more severe portal hypertension. In this context, bleeding control becomes more difficult, the risk of rebleeding increases, and patients are more prone to progression to multi-organ dysfunction. In our study, higher Child–Pugh class and the presence of HCC were independently associated with 6-week mortality, while HCC was also associated with 6-week rebleeding. These findings are consistent with previous reports. Cho et al. demonstrated that Child–Pugh class C was an independent predictor of rebleeding and mortality, and HCC was also associated with increased mortality [22]. Similarly, Wang et al. reported that higher Child–Pugh class, bilirubin, and creatinine levels were associated with an increased risk of 6-week rebleeding [23]. Wakatsuki et al. found that Child–Pugh class B/C and HCC were associated with increased mortality in patients with gastric varices [24]. Nguyen et al. further identified Child–Pugh class C as one of the strongest predictors of in-hospital mortality [12]. These findings underscore the importance of incorporating liver function and tumor status into risk stratification and clinical decision-making. The Baveno VII consensus recommends more aggressive management strategies for patients with Child–Pugh class C bleeding [1], and early TIPS has been shown to improve outcomes in this high-risk population [25].

With respect to secondary outcomes, endoscopy timing was associated with length of hospital stay and transfusion requirements. Compared with the early group (< 24 h), patients in the delayed group (≥ 72 h) had a significantly longer hospital stay, whereas no significant difference was observed between the intermediate and early groups. These findings suggest that delayed endoscopy may be associated with a longer hospitalization course, possibly reflecting prolonged stabilization, delayed procedural scheduling, or greater clinical complexity. Similar observations have been reported in non-variceal upper gastrointestinal bleeding, where delayed endoscopy has been associated with prolonged hospital stay [26]. Regarding transfusion requirements, patients in the intermediate group had significantly lower transfusion volume compared with those in the early group in the multivariable model. This may reflect differences in initial clinical acuity and resuscitation needs across timing groups. Patients in the early group had a higher proportion of ICU admission, suggesting that they may have had more severe acute presentations or required more intensive monitoring and support before or during early endoscopic intervention. Therefore, the lower transfusion volume observed in the intermediate group should not be interpreted as evidence of a lower-risk clinical course. This pattern may also reflect different clinical trajectories, possible delayed recognition of severity, or variation in early resuscitation intensity among patients who initially appeared clinically stable.

In addition to timing, baseline patient characteristics also significantly influenced secondary outcomes. Lower serum albumin levels were associated with longer hospital stay, higher hospitalization costs, and increased transfusion requirements. Albumin plays a key role in maintaining intravascular volume, molecular transport, and immune and antioxidant functions. In patients with cirrhosis, both the quantity and functional capacity of albumin are reduced, contributing to disease progression [27]. Previous studies have shown that albumin supplementation in cirrhotic patients with acute gastrointestinal bleeding is associated with reduced rebleeding and mortality [28]. Furthermore, albumin therapy may improve circulatory function and reduce systemic inflammation, providing a potential mechanism for improved outcomes [29].

In addition, higher Child–Pugh class was associated with increased transfusion requirements. Patients with advanced cirrhosis often have impaired coagulation and persistent portal hypertension, making hemostasis more difficult and increasing dependence on blood transfusion. Previous studies have reported that patients with Child–Pugh class C are more likely to require massive transfusion [30].

This study has several limitations. First, as a single-center retrospective study, selection bias and information bias cannot be completely excluded.

Second, the timing of endoscopy was determined by the treating physicians and clinical workflow rather than random allocation; therefore, confounding by indication may persist, and the observed associations should not be interpreted as evidence of a causal effect of endoscopy timing on clinical outcomes. Endoscopy timing was measured from hospital admission rather than bleeding onset, which may limit comparability with guideline-based definitions and previous studies. Although ICU admission and pre-endoscopy transfusion volume were incorporated into the revised analyses, these variables are only indirect markers and cannot fully capture acute bleeding severity or physiologic instability, such as shock, detailed hemodynamic status, lactate levels, vasopressor requirement, requirement for endotracheal intubation, active bleeding at endoscopy, or the exact time from bleeding onset to endoscopy. In addition, the specific reasons for delayed endoscopy, including patient condition, resource availability, procedural scheduling, or physician decision-making, as well as the presence of ongoing bleeding before endoscopy, could not be reliably classified from the retrospective records. Third, the distinction between esophageal and gastric variceal bleeding could not be fully analyzed, and endoscopic therapy differed across timing groups. Although endoscopic treatment modalities were summarized, treatment selection may reflect lesion characteristics and clinical judgment and may contribute to residual confounding. Fourth, the number of mortality events was small, resulting in wide confidence intervals despite the use of Firth penalized logistic regression. Therefore, the mortality estimates had limited precision and should be interpreted as exploratory rather than definitive; even a small change in the number of events, particularly in the reference group, could materially affect the estimated odds ratios. Fifth, although IPTW was performed as a sensitivity analysis and the weighted models were additionally adjusted for age because of residual age imbalance, residual confounding could not be fully eliminated. Finally, this study focused on 6-week outcomes and did not assess longer-term outcomes, such as recurrent bleeding, overall survival, transplant-free survival, or quality of life.

## Conclusion

In conclusion, endoscopy timing is closely associated with short-term outcomes in patients with AVB. Endoscopy performed at 24–72 h was associated with higher odds of 6-week mortality and rebleeding compared with endoscopy performed within 24 h, whereas the association between endoscopy beyond 72 h and adverse outcomes was less consistent across sensitivity analyses. Liver function status and the presence of HCC were also important determinants of prognosis. These findings are consistent with the importance of timely endoscopy after adequate resuscitation and stabilization, while emphasizing the need for individualized management based on patient condition and disease severity.

## Supplementary Information

Supplementary Material 1.

## Data Availability

No datasets were generated or analysed during the current study.

## Abbreviations

AIH: Autoimmune hepatitis
AUGIB: Acute upper gastrointestinal bleeding
AVB: Acute variceal bleeding
CI: Confidence interval
EIS: Endoscopic injection sclerotherapy
EVL: Endoscopic variceal ligation
EVO: Endoscopic variceal obturation
HCC: Hepatocellular carcinoma
ICU: Intensive care unit
INR: International normalized ratio
IQR: Interquartile range
IPTW: Inverse probability of treatment weighting
MELD: Model for End-Stage Liver Disease
aOR: Adjusted odds ratio
PT: Prothrombin time
