# Supplementary material for: Timing of endoscopy and short-term outcomes in cirrhotic patients with acute variceal bleeding: a single-center retrospective study
Source: BMC Gastroenterol. 2026 Jul 20;26:644. doi: 10.1186/s12876-026-05133-5 (PMC13663163; doi:10.1186/s12876-026-05133-5)
Supplement: Supplementary file 1 [file 12876_2026_5133_MOESM1_ESM.docx]

Supplementary Table 1. Endoscopic treatment modalities according to endoscopy timing

| **Endoscopic treatment modality** | **All patients** | **Early endoscopy (<24 h)** | **Intermediate endoscopy (24–72 h)** | **Delayed endoscopy (≥72 h)** | **P value** |
| --- | --- | --- | --- | --- | --- |
|  | **N = 213** | **N = 45** | **N = 55** | **N = 113** |  |
| EVL | 25 (11.7%) | 6 (13.3%) | 5 (9.1%) | 14 (12.4%) | 0.782 |
| EIS | 175 (82.2%) | 35 (77.8%) | 47 (85.5%) | 93 (82.3%) | 0.588 |
| EVO | 61 (28.6%) | 17 (37.8%) | 20 (36.4%) | 24 (21.2%) | **0.039** |
| Combined endoscopic therapy | 48 (22.5%) | 13 (28.9%) | 17 (30.9%) | 18 (15.9%) | **0.046** |

Supplementary Table 2. Primary outcomes according to endoscopy timing in the non-ICU subgroup

| **Clinical outcomes** | **All non-ICU patients** | **Early endoscopy (<24 h)** | **Intermediate endoscopy (24–72 h)** | **Delayed endoscopy (≥72 h)** | **P value** |
| --- | --- | --- | --- | --- | --- |
|  | **N = 171** | **N = 20** | **N = 50** | **N = 101** |  |
| **6-week mortality** | 12 (7.0%) | 0 (0%) | 7 (14%) | 5 (5.0%) | 0.071 |
| **6-week rebleeding** | 45 (26.3%) | 3 (15%) | 17 (34%) | 25 (24.8%) | 0.254 |

Supplementary Table 3. Full sensitivity models for primary outcomes additionally adjusted for pre-endoscopy transfusion volume

|  | **6-week mortality**  **aOR (95% CI)** | **P value** | **6-week rebleeding**  **aOR (95% CI)** | **P value** |
| --- | --- | --- | --- | --- |
| **Timing of endoscopy** |  |  |  |  |
| <24 h (reference) | Reference | — | Reference | — |
| 24–72 h | 23.2 (2.59–438) | **0.003** | 3.29 (1.07–11.1) | **0.044** |
| ≥72 h | 11.0 (1.17–210) | **0.035** | 2.27 (0.76–7.49) | 0.156 |
| **Age (years)** | 1.05 (1.00–1.10) | 0.076 | 1.03 (1.00–1.06) | 0.071 |
| **Child-Pugh class (per class increase)** | 11.5 (2.33–66.2) | **0.002** | 1.48 (0.71–3.14) | 0.302 |
| **Hepatocellular carcinoma (Yes vs No)** | 9.56 (2.96–36.8) | **<0.001** | 3.43 (1.61–7.36) | **0.001** |
| **ICU admission (Yes vs No)** | 3.51 (0.67–17.1) | 0.131 | 1.21 (0.42–3.36) | 0.721 |
| **Pre-endoscopy transfusion (units)** | 0.97 (0.77–1.16) | 0.744 | 1.00 (0.88–1.11) | 0.935 |

Supplementary Table 4. Full sensitivity models for primary outcomes using MELD score instead of Child–Pugh class

|  | **6-week mortality**  **aOR (95% CI)** | **P value** | **6-week rebleeding**  **aOR (95% CI)** | **P value** |
| --- | --- | --- | --- | --- |
| **Timing of endoscopy** |  |  |  |  |
| <24 h (reference) | Reference | — | Reference | — |
| 24–72 h | 12.0 (1.71–161) | **0.010** | 3.52 (1.17–11.7) | **0.031** |
| ≥72 h | 4.16 (0.60–53.2) | 0.158 | 2.14 (0.77–6.61) | 0.162 |
| **Age (years)** | 1.04 (0.99–1.10) | 0.096 | 1.03 (1.00–1.06) | **0.042** |
| **MELD score** | 1.12 (0.95–1.26) | 0.139 | 1.09 (0.99–1.19) | 0.064 |
| **Hepatocellular carcinoma (Yes vs No)** | 8.55 (2.86–28.4) | **<0.001** | 3.19 (1.53–6.68) | **0.002** |
| **ICU admission (Yes vs No)** | 3.41 (0.65–15.9) | 0.138 | 1.26 (0.46–3.29) | 0.636 |

Supplementary Table 5. Balance diagnostics after inverse probability of treatment weighting

| **Variable** | **Before IPTW SMD** | **After IPTW SMD** | **Balance after IPTW** |
| --- | --- | --- | --- |
| **Age** | 0.153 | 0.220 | Imbalanced |
| **Sex** | 0.318 | 0.079 | Good |
| **MELD score** | 0.417 | 0.181 | Acceptable |
| **HCC** | 0.162 | 0.037 | Good |
| **ICU admission** | 1.215 | 0.043 | Good |
| **Antibiotic use** | 0.183 | 0.096 | Good |

The IPTW analysis included patients with complete covariate data for the propensity score model. SMD <0.1 was considered good balance, 0.1–0.2 acceptable balance, and >0.2 imbalance.

Supplementary Table 6. IPTW-weighted analyses of primary outcomes

| **Clinical outcomes** | **Comparison** | **OR (95% CI)** | **P value** |
| --- | --- | --- | --- |
| **6-week mortality** | 24–72 h vs <24 h | 16.32 (1.88–141.48) | **0.012** |
| **6-week mortality** | ≥72 h vs <24 h | 6.55 (0.70–61.35) | 0.101 |
| **6-week rebleeding** | 24–72 h vs <24 h | 3.27 (1.02–10.48) | **0.048** |
| **6-week rebleeding** | ≥72 h vs <24 h | 2.17 (0.73–6.49) | 0.166 |

IPTW-weighted outcome models were additionally adjusted for age because residual age imbalance remained after weighting.

Supplementary Table 7. Primary outcomes according to guideline-based four-category endoscopy timing groups

| **Clinical outcomes** | **All patients  N = 213** | **≤12 h  N = 17** | **12–24 h  N = 28** | **24–72 h  N = 55** | **≥72 h  N = 113** | **P value** |
| --- | --- | --- | --- | --- | --- | --- |
| **6-week mortality** | 15 (7.0%) | 0 (0%) | 1 (3.6%) | 8 (15%) | 6 (5.3%) | 0.12 |
| **6-week rebleeding** | 54 (25%) | 3 (18%) | 4 (14%) | 19 (35%) | 28 (25%) | 0.2 |

Supplementary Table 8. Full multivariable models for primary outcomes using guideline-based four-category endoscopy timing groups

|  | **6-week mortality**  **aOR (95% CI)** | **P value** | **6-week rebleeding**  **aOR (95% CI)** | **P value** |
| --- | --- | --- | --- | --- |
| **Timing of endoscopy** |  |  |  |  |
| ≤12 h (reference) | Reference | — | Reference | — |
| 12–24 h | 9.96 (0.30–1959.72) | 0.203 | 0.95 (0.17–5.82) | 0.954 |
| 24–72 h | 72.40 (3.27–14519.12) | **0.004** | 3.24 (0.74–18.04) | 0.139 |
| ≥72 h | 33.26 (1.47–6638.14) | **0.024** | 2.17 (0.53–11.61) | 0.314 |
| **Age (years)** | 1.05 (1.00–1.10) | 0.078 | 1.03 (1.00–1.06) | 0.075 |
| **Child-Pugh class (per class increase)** | 14.26 (2.75–88.91) | **0.001** | 1.56 (0.76–3.26) | 0.230 |
| **Hepatocellular carcinoma (Yes vs No)** | 11.81 (3.42–51.82) | **<0.001** | 3.23 (1.53–6.86) | **0.002** |
| **ICU admission (Yes vs No)** | 3.14 (0.59–15.81) | 0.172 | 1.19 (0.43–3.11) | 0.726 |

Supplementary Table 9. Primary outcomes according to simplified guideline-based three-category endoscopy timing groups

| **Clinical outcomes** | **All patients  N = 213** | **≤12 h  N = 17** | **12–24 h  N = 28** | **>24 h  N = 168** | **P value** |
| --- | --- | --- | --- | --- | --- |
| **6-week mortality** | 15 (7.0%) | 0 (0%) | 1 (3.6%) | 14 (8.3%) | 0.5 |
| **6-week rebleeding** | 54 (25%) | 3 (18%) | 4 (14%) | 47 (28%) | 0.3 |

Supplementary Table 10. Full multivariable models for primary outcomes using simplified guideline-based three-category endoscopy timing groups

|  | **6-week mortality**  **aOR (95% CI)** | **P value** | **6-week rebleeding**  **aOR (95% CI)** | **P value** |
| --- | --- | --- | --- | --- |
| **Timing of endoscopy** |  |  |  |  |
| ≤12 h (reference) | Reference | — | Reference | — |
| 12–24 h | 11.22 (0.33–2203.73) | 0.181 | 0.95 (0.17–5.84) | 0.954 |
| >24 h | 51.36 (2.42–9992.30) | **0.007** | 2.50 (0.62–13.14) | 0.226 |
| **Age (years)** | 1.05 (1.00–1.10) | 0.070 | 1.03 (1.00–1.05) | 0.082 |
| **Child-Pugh class (per class increase)** | 15.84 (3.13–98.61) | **<0.001** | 1.59 (0.78–3.33) | 0.207 |
| **Hepatocellular carcinoma (Yes vs No)** | 13.59 (3.95–59.98) | **<0.001** | 3.31 (1.58–6.99) | **0.002** |
| **ICU admission (Yes vs No)** | 3.05 (0.56–15.40) | 0.185 | 1.18 (0.43–3.06) | 0.742 |
